# Autoantibody hotspots reveal the origin and impact of immunogenic XIST ribonucleoprotein complexes in autoimmune diseases

**DOI:** 10.1172/JCI198291

**Published:** 2026-02-10

**Authors:** Bingyu Yan, Jinwoo Lee, Suhas Srinivasan, Pedro Ambriz, Quanming Shi, Diana R. Dou, Srijana Davuluri, Swarna Nandyala, Adrianne Woods, Gwendolyn Leatherman, Yanding Zhao, Roman E. Reggiardo, Manasi Sawant, Hawa Racine Thiam, Ami A. Shah, David F. Fiorentino, Lorinda S. Chung, Howard Y. Chang

**Affiliations:** 1 Department of Dermatology and; 2 Division of Immunology and Rheumatology, Department of Medicine, Stanford University School of Medicine, Stanford, California, USA.; 3 RNA Medicine Program, Stanford University, Stanford, California, USA.; 4 Division of Rheumatology, Johns Hopkins University School of Medicine, Baltimore, Maryland, USA.; 5 Department of Bioengineering and; 6 Department of Microbiology and Immunology, Stanford University, Stanford, California, USA.; 7 Department of Genetics, Stanford University School of Medicine, Stanford, California, USA.

**Keywords:** Autoimmunity, Immunology, Inflammation, Antigen, Autoimmune diseases, Biomarkers

## Abstract

Novel autoantibodies target hotspots on the XIST ribonucleoprotein complex in female-biased autoimmune diseases.

**To the Editor:** Four out of five patients with autoimmune diseases are women, but standard autoantibody testing has used male cells for over 40 years (1). XIST, a female-specific long noncoding RNA responsible for X chromosome inactivation, forms large ribonucleoprotein (RNP) complexes that comprise over 100 proteins and promote female-biased autoimmunity (2). Here, we identify autoantibody hotspots within XIST RNPs and demonstrate that antibody reactivity against XIST RNP components is clinically relevant in systemic sclerosis (SSc), linking anti-XIST RNP autoantibodies to systemic autoimmunity.

We analyzed XIST-associated proteins (XAPs), previously mapped through RNA-binding proteomics (3, 4), and by correlating autoantibody targets in autoimmune patient sera with their occupancy sites on XIST (Supplemental Figure 1, C and D), we show that XAP autoantigens are guided by RNA secondary structure and concentrated at discrete domains along the 19 kb XIST RNA, particularly the A-repeat (Figure 1A). These findings suggest that the structural organization of the XIST complex creates immunogenic hotspots prone to self-recognition. Notably, SPEN (and a related protein, RBM15) emerged as a major antigenic target, consistent with its role as a core A-repeat–binding factor in the XIST complex (5).

To investigate potential cellular sources of hotspot antigens, we examined publicly available immune cell protein expression datasets, which indicated neutrophils as a predominant source of hotspot antigens among blood cells (Figure 1B). NETosis is an immunogenic pathway upon which neutrophils extrude their nuclear content in neutrophil extracellular traps (NETs). We imaged human neutrophils and found that upon induction of NETosis, the proteins SPEN and RBM15, along with XIST RNA, were distributed to extracellular DNA traps (Figure 1C and Supplemental Figure 2B; supplemental material available online with this article; https://doi.org/10.1172/JCI198291DS1 These data identify NETosis as a plausible mechanism for externalizing nuclear XIST RNP antigens.

In the pristane-induced mouse model of autoimmunity, sera collected during disease onset showed increased recognition of XAPs (2), supporting the immunogenicity of these complexes in vivo. To determine clinical relevance, we assessed anti-SPEN seropositivity in a cohort of patients with SSc (Figure 1D) and found that patients with high levels of autoantibodies against SPEN exhibited a higher frequency of severe digital ischemia and vasculopathy (Figure 1, E and F). Because of the small size of the Stanford patient cohort, an inherent limitation in the investigation of rare diseases, statistical significance was only seen for the clinical outcome of digital gangrene/amputation (difference in proportion = 0.40, 95% CI = 0.09–0.70, P = 0.0088, Barnard’s exact test; Figure 1E). Differences trending toward but not reaching statistical significance could also be seen for the pathologically related outcome of digital ulcers (difference in proportion = 0.23, 95% CI = –0.16–0.55, P = 0.25, Barnard’s exact test). We sought to validate this finding in an independent cohort, with analysis performed by independent investigators. In a larger cohort of 35 patients with SSc from Johns Hopkins University (JHU), we found a statistically significant association between high anti-SPEN reactivity and severe Raynaud’s phenomenon (Supplemental Figure 2C; difference in proportion = 0.37, 95% CI = 0.047–0.60, P = 0.031, Barnard’s exact test), which was defined by the JHU investigators as digital pitting scars, digital tip ulceration, and digital gangrene (Supplemental Figure 2C). In addition, we found a statistically significant association between high anti-SPEN antibody and digital ulceration (difference in proportion = 0.33, 95% CI = 0.0033–0.60, P = 0.044, Barnard’s exact test; Supplemental Figure 2C). Clinical features of SPEN-positive patients are summarized in Supplemental Table 1.

Our data suggest a mechanistic link between XIST RNP structure, neutrophil biology, and female-biased autoimmunity (Figure 1G). By defining autoantibody hotspots within the XIST RNA protein scaffold and demonstrating their release during NETosis, we identified a plausible mechanism that exposes XIST to B cells and autoantibody formation. These findings highlight anti-XAP autoantibodies as potential biomarkers and functional entry points for understanding sex differences in human immunity.

For detailed methods, information regarding sex as a biological variable, statistics, study approval, and author contributions, see the supplemental materials.

## Funding support

This work is the result of NIH funding, in whole or in part, and is subject to the NIH Public Access Policy. Through acceptance of this federal funding, the NIH has been given a right to make the work publicly available in PubMed Central.

- NIH grants U54 AR085970 (LSC, HYC), K24 AR080217 (AAS), R01 AR073208 (AAS), and P30 AR070254 (AAS).
- Scleroderma Research Foundation (JL, HYC).
- Stanford RNA Medicine Program (HYC).
- Howard Hughes Medical Institute (HYC).
- Stanford School of Medicine Dean’s Postdoctoral Fellowship (BY).
- Dermatology Foundation (JL).
- American Skin Association (JL).
- Donald B. and Dorothy L. Stabler Foundation (AAS).
- Sara and Alex Othon Research Fund (AAS).
- Chresanthe Staurulakis Memorial Fund (AAS).

## Supplementary Material

Supplemental data

Supporting data values

## Figures and Tables

**Figure 1:**
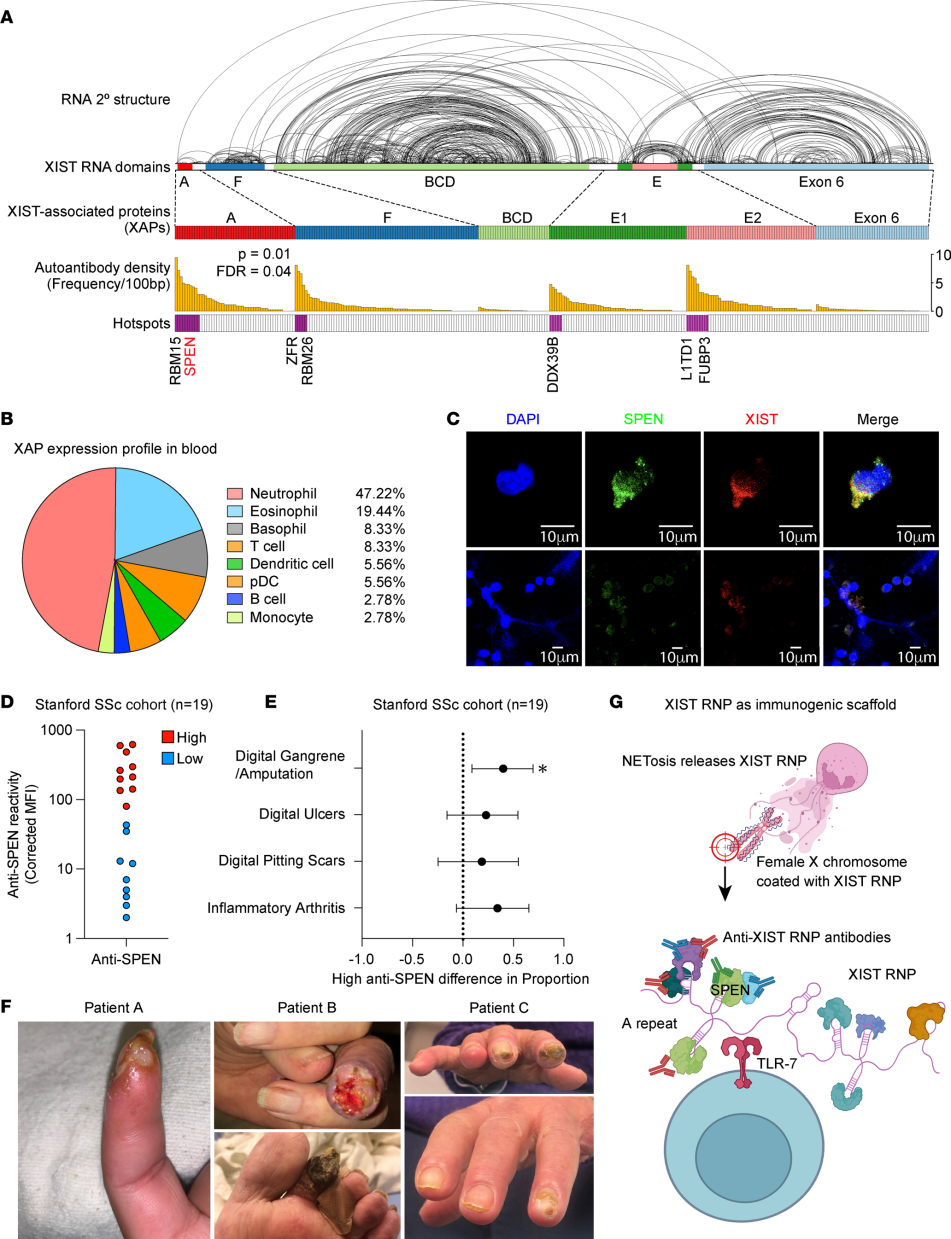
Immunogenic XAPs are released by NETosis and associated with high-risk vasculopathy in patients with SSc. (**A**) Binding profile for XAPs. XIST secondary structure was determined by psoralen cross-linking in living cells and deep sequencing (3); each arc on top represents an RNA duplex along XIST RNA. Clusters of XAP are indicated by bars that are color coded to XIST functional domains. Frequency of autoreactivity (MFI > 100) to XAP-derived autoantigens in patients with autoimmune disease is indicated by orange bars. Hotspots that are significantly elevated in any autoimmune disease are indicated by purple bars; others are indicated by white bars. (**B**) Cell of origin for antigenic proteins. Proportions for each cell type are displayed as a pie chart. (**C**) dHL-60 cells were stimulated with ionomycin to induce NETosis and stained with DAPI (blue), anti-SPEN (green), and XIST FISH probe (red). Scale bars: 10 μm. (**D**) Distribution of MFI values for sera reactivity against SPEN in the Stanford scleroderma cohort. (**E**) Calculated ORs for categorical variables of interest in the Stanford scleroderma cohort as defined by Stanford investigators, with horizontal lines indicating the 95% CIs, and a dotted vertical line at 1.0 signifying no association. *P < 0.05. P values were calculated using Barnard’s unconditional exact test. (**F**) Representative clinical photos of severe digital ulceration and gangrene in 3 patients with high anti-SPEN sera reactivity. (**G**) Model of the XIST RNP complex acting as an immunogenic scaffold. Created in BioRender.

## Version 1 02/10/2026

In-Press Preview

## Version 2 04/01/2026

Electronic publication
