# Supplementary material for: Autoantibody hotspots reveal the origin and impact of immunogenic XIST ribonucleoprotein complexes in autoimmune diseases
Source: J Clin Invest. 2026 Feb 10;136(7):e198291. doi: 10.1172/JCI198291 (PMC13038191; doi:10.1172/JCI198291)
Supplement: Supplemental data [file jci-136-198291-s157.pdf]

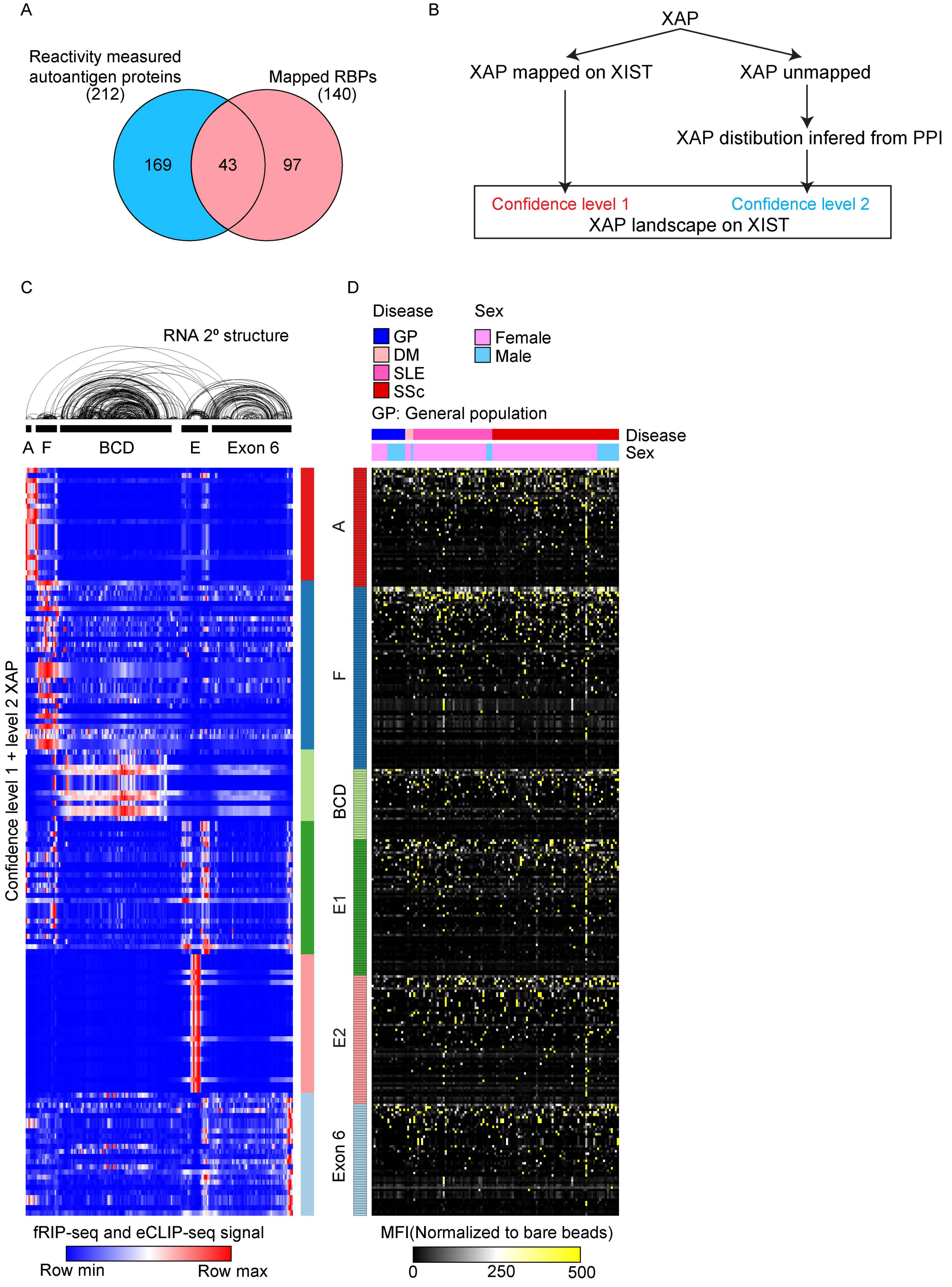
A
Reactivity measured autoantigen proteins (212)
Mapped RBPs (140)
169
43
97
B
XAP
XAP mapped on XIST
XAP unmapped
XAP distibution infered from PPI
Confidence level 1
Confidence level 2
XAP landscape on XIST
C
RNA 2º structure
A F BCD E Exon 6
Confidence level 1 + level 2 XAP
fRIP-seq and eCLIP-seq signal
Row min
Row max
D
Disease
GP
DM
SLE
SSc
GP: General population
Sex
Female
Male
Disease
Sex
A
F
BCD
E1
E2
Exon 6
MFI(Normalized to bare beads)
0
250
500

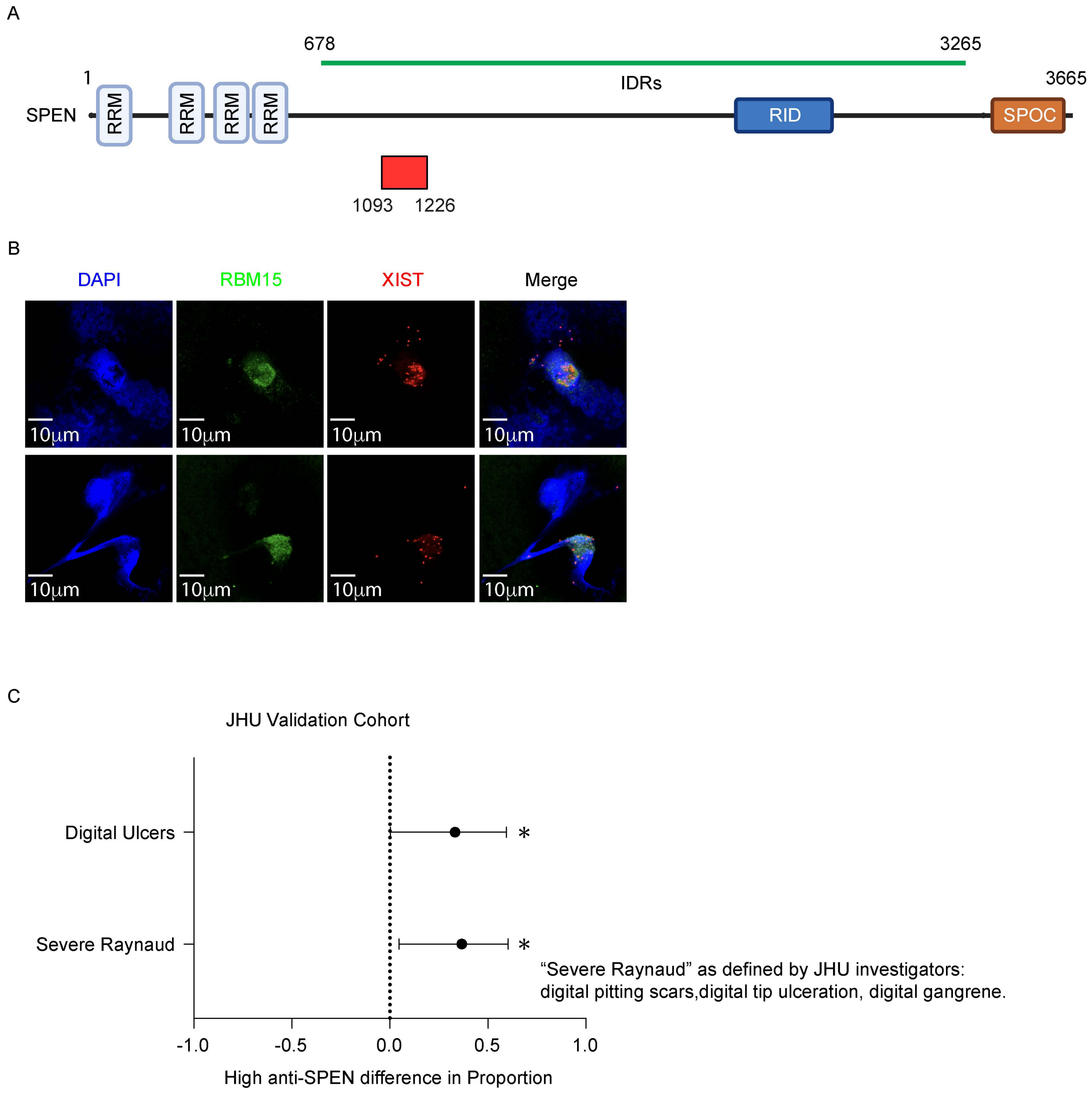
A
678
3265
IDRs
1
3665
SPEN
RRM
RRM
RRM
RRM
RID
SPOC
1093
1226
B
DAPI
RBM15
XIST
Merge
10μm
C
JHU Validation Cohort
Digital Ulcers
Severe Raynaud
*
*
"Severe Raynaud" as defined by JHU investigators:
digital pitting scars,digital tip ulceration, digital gangrene.
-1.0
-0.5
0.0
0.5
1.0
High anti-SPEN difference in Proportion

Supplementary Figure legends:

Figure S1: Immunogenicity of XAPs is associated with XIST functional domains. (**A**) Overlap of reactivity quantified antigenic proteins with XIST-associated proteins with available location information. Number of reactivity-quantified antigenic proteins with available location information, displayed as intersection from the Venn diagram. **(B)** Workflow for assigning and inferring the location of antigenic proteins in XIST RNA by protein-protein interaction. (**C**) Binding profile for XIST-associated proteins (XAP). Row normalized binding intensity for each XAP are displayed as heatmap in each row within 100-bp windows across XIST RNA. XIST secondary structure was determined by psoralen crosslinking in living cells and deep sequencing(7); each arc on top represents a RNA duplex along XIST RNA. Clusters of XAP are indicated by the bar on the right-hand side. **(D)** Reactivity of XAP derived autoantigens across general population and autoimmune disease patients. Disease annotations and sex annotations are on the top of the heatmap. Annotations for XAP-derived autoantigens in different XIST functional domains are shown on two sides of the heatmap.

Figure S2: AXA hotspot is associated with high-risk vasculopathy in systemic sclerosis patients. (A) Schematic showing the location of the protein fragment used in the fluorescent bead-based antigen array to assess sera reactivity against SPEN. RRM, RNA recognition motif. RID, receptor interaction domain. SPOC, SPEN paralog and ortholog C-terminal. IDRs, intrinsically disordered regions. (**B**) Human neutrophils were stimulated with PMA to induce NETosis and stained with DAPI (blue), anti-RBM15 (green), and XIST FISH probe (red). **(C)** Calculated odds ratios for digital ulcers and severe Raynaud phenomenon (*i.e.* digital pitting scars, digital tip ulceration, digital gangrene) in the JHU validation scleroderma cohort, as defined by JHU investigators. * $p < 0.05$.

Supplementary method and materials

Sex as a biological variable

Our study examined both males and females, and the nature of our work focuses on the consideration of sex as a biological variable.

Protein-protein interaction analysis

Proteins comprising autoantigens were first mapped to XIST RBPs which have fRIP-seq or eCLIP-seq data by physical protein-protein interaction from multiple databases by Metascape (19). The proteins that comprised the remaining autoantigens and unassigned were mapped to other RBPs capable of binding XIST from the fRIP-seq or eCLIP-seq data. The interacting partner with the highest score was kept in the analysis. Confidence level 1 interactions represent direct XIST–protein associations confirmed by eCLIP or fRIP data; confidence level 2 interactions represent indirect protein–protein interactions mapped via curated databases (**Supplemental Figure 1A and B**).

With the XAPs assigned onto the XIST RNA sequence, *k*-means clustering was performed with values from fRIP-seq or eCLIP-seq data, and these clusters were assigned to XIST functional domains (**Supplemental Figure 1C**).

AXA hotspot identification

The reactivity of AXA against each XAP autoantigen with normalized (AXA - bare beads) MFI was projected on XIST (**Supplemental Figure 1D**). The number of individuals with reactivity of a particular AXA with normalized (AXA - bare beads) MFI>100 is counted. Top 10% of the autoantigens are defined as AXA hotspots in human. All the autoantibody reactivity data were obtained from (2). In this study, 'autoantibody reactivity' refers to the quantitative signal

intensity of antigen–antibody binding measured by suspension bead array, whereas 'immunogenicity' denotes the population-level prevalence of high-titer responses across individuals, reflecting the antigen's propensity to elicit immune recognition. Autoantibody density was calculated by counting the number of patients who have reactivity MFI > 100 and being divided by the number of 100bp windows within each XIST domain.

Human protein atlas

Data were acquired from the Human Protein Atlas (www.proteinatlas.org) (11). The blood profile of all proteins was retrieved and matched to XIST-associated proteins to indicate the main cell types that contributed to protein expression in blood.

Neutrophil isolation

Human neutrophils were isolated from the blood of healthy donors collected by the Research and Clinical Services team at the Stanford Blood Center or by customized collection from a commercial vendor (Creative Biolabs). When isolation was performed from whole blood in our laboratory and not from a commercial vendor, we performed density gradient-based cell separation, described as follows. In brief, 7 ml of heparinized blood was layered on 7 ml of Histopaque-1119 (Sigma-Aldrich, 11911) in a 15-ml Falcon tube, and centrifuged at 800 × *g* for 20 minutes at room temperature. The upper layer and interphase were then aspirated and discarded, while the diffuse red phase above the erythrocyte pellet was collected in a new tube, washed with HBSS, then layered onto a 10-ml density gradient consisting of successive layers of 65, 70, 75, 80, and 85% Percoll (Sigma-Aldrich, P4937) and centrifuged at 800 × *g* for 20 minutes at room temperature. The interface between the 65% and 75% Percoll layers were collected in a new 15-ml tube, washed, then resuspended in RPMI at 1.5×10^6 cells per ml for neutrophil activation.

Human neutrophil activation

Human neutrophils were stimulated by phorbol myristate acetate (PMA) as previously described (20). In brief, isolated neutrophils were incubated for 1-3 hours with 30 nM PMA (Sigma-Aldrich, P8319) at 37 °C, then fixed with 1% formaldehyde for 10 minutes at room temperature and spun down onto a glass slide with Cytospin at 1000 r.p.m. for 5 minutes at room temperature for FISH and immunostaining.

HL-60 cells

HL-60 cells (ATCC CCL-240) were cultured at 37 °C and 5% CO2 in RPMI 1640 plus L-glutamine with 25 mM HEPES buffer, 1% penicillin/streptomycin, and 15% heat-inactivated FBS and differentiated to neutrophil-like cells by addition of 1.3% of DMSO. For NETosis, HL-60 cells were stimulated with ionomycin (4 μM) for 2 h.

FISH and immunofluorescence imaging

After fixation, slides were washed with PBS, then Stellaris™ RNA FISH Probes recognizing XIST and labelled with Quasar™ 570 dye (SMF-2038-1, LGC, Biosearch Technologies, Petaluma, CA) were hybridized to samples, following the manufacturer's instructions available online at www.biosearchtech.com/stellarisprotocols. FISH and IF were performed according to the manufacturer-provided protocol without any significant deviations. Briefly, after allowing slides to air dry following cell permeabilization with 70% ethanol for 1 hour at 4 °C, slides were immersed in Stellaris® RNA FISH Wash Buffer A (SMF-WA1) for 2-5 minutes at room temperature. Then, slides were hybridized overnight for 16 hours in a light-protected humidified chamber at 37 °C in 125 nM of Human XIST Stellaris® FISH Probes with Quasar® 570 Dye (SMF-2038-1) hybridization buffer containing the probe and appropriately diluted primary

antibody. After washing, the slides were then incubated with appropriately diluted secondary antibody in Wash Buffer A for 30 minutes in a light-protected chamber at 37 °C, followed by immersion in Stellaris® RNA FISH Wash Buffer B (SMF-WB1) for 2-5 minutes at room temperature. Slides were mounted with Vectashield Mounting Medium with DAPI (Vector Laboratories, H-1800) and a cover glass.

Slides were imaged at Stanford University - CSIF: Cell Sciences Imaging Facility, RRID:SCR_017787 on the Leica Stellaris 8 DIVE, with data acquisition using LAS X software.

Primary antibodies were used as follows: rabbit anti-SPEN (1:250, Abcam, ab72266), rabbit anti-RBM15 (1:100, Proteintech, 10587-1-AP).

Secondary antibodies we used as follows: goat anti-rabbit-AF488 (1:400, Thermo Fisher, A11008) or goat anti-mouse-AF647 (1:400, Thermo Fisher, A-21244), goat anti-mouse-AF488 (1:400, Thermo Fisher, A-32723).

XIST autoantigen array analysis

Fragments of XIST-associated proteins were chosen as previously described and collected patient sera were assayed by bead array as previously reported(2). In brief, samples were prepared by loading 25 µl of randomized patient serum or blank control onto a 96-well plate, which was subsequently shipped to SciLifeLab (Sweden) for suspension bead array assay. Non-specific reactivity was adjusted for by subtracting the MFI signal of “bare beads” from the raw MFI signals of patient samples. To compare groups with high and low reactivities, percentile ranks were used within cohorts to avoid batch effects.

Study approval

All human patient samples were de-identified in this study and obtained under the respective institutions' IRB-approved protocols. Sera from systemic sclerosis patients in the Stanford cohort were collected as previously published under IRB #12047 (2). Sera from systemic sclerosis patients in the Johns Hopkins cohort were collected as previously published under NA_00039566 and IRB00226995 (2).

Data availability

All publicly available data used in this study are cited in the text. All data generated by this study are included in the Supplemental data and Supporting data values sections.

Statistics

All analyses were performed using R Statistical Software (v4.4.1; R Core Team 2023). Unconditional exact tests were performed using the Exact (Unconditional Exact Test) package (v3.3; (21)) using *exact.test* with the CSM (Convexity, Symmetry, and Minimization) method.

Supplemental Table 1. Details of scleroderma patient cohorts.

Characteristic	Stanford (N = 24)	Johns Hopkins (N = 35)
**Sex**		
- **Female**	22	27
- **Male**	2	8
**Race**		
- **White**	15	29
- **Black**	1	3
- **Asian**	6	2
- **Middle Eastern**	0	1
- **Mixed or other**	2	0
**SSc subtype**		
- **Diffuse**	9	13
- **Limited**	15	22
